# Study protocol for optimising antipsychotic prescribing among hospitalised patients in the acute care setting in Scotland: a national retrospective cohort study

**DOI:** 10.1136/bmjopen-2025-098927

**Published:** 2025-12-10

**Authors:** Cosmika Goswami, Tanja Mueller, Alexa Wall, Chris F Johnson, David Grosset, Marion Bennie, Amanj Kurdi

**Affiliations:** 1 Strathclyde Institute of Pharmacy and Biomedical Sciences, University of Strathclyde, Glasgow, UK; 2 Pharmacy Services, National Health Services Lothian, Edinburgh, UK; 3 Pharmacy Services, National Health Services Greater Glasgow & Clyde, Glasgow, UK; 4 Psychiatry, National Health Services Lothian, Edinburgh, UK; 5 College of Pharmacy, Al-Kitab University, Kirkuk, Iraq; 6 Department of Public Health Pharmacy and Management, School of Pharmacy, Sefako Makgatho Health Sciences University, Pretoria, South Africa

**Keywords:** MENTAL HEALTH, INTENSIVE & CRITICAL CARE, Polypharmacy

## Abstract

**Abstract:**

**Introduction:**

Prescribing high-dose antipsychotics is typically reserved for individuals with treatment-resistant severe mental illnesses, such as schizophrenia, bipolar disorder and psychotic depression. It carries an increased risk of adverse drug effects, necessitating regular monitoring. Non-mental health specialist clinicians may not always be aware when the maximum recommended dose of antipsychotics is exceeded, leading to unintentional high-dose prescribing without recognising the need for additional monitoring or understanding the associated risks. Therefore, providing clinical decision support (CDS) tools to support clinicians and improve the appropriate prescribing of antipsychotics is important. The aim of this study is to understand current prescribing practices and assess the impact of high-dose antipsychotic prescribing on clinical outcomes among hospitalised patients. The findings from this study will shape a future project focused on developing an integrated computerised CDS tool.

**Methods and analysis:**

This retrospective cohort study will examine antipsychotic prescribing among hospitalised patients using Hospital Electronic Prescribing and Medicines Administration data in Scotland from 2019 to 2023, in linkage with hospital records, Scottish Morbidity Records and primary care prescribing (Prescribing Information System). Patients will be grouped into those prescribed high-dose (exposed), defined as exceeding the 100% maximum recommended British National Formulary dose and normal-dose (unexposed) antipsychotics, followed from their first ever antipsychotic prescription date (index date) until the end of the study, study outcomes or death, whichever happens first. We will quantify high-dose antipsychotic prescribing, profile patient characteristics and use machine learning techniques to assess associations of high-dose antipsychotic prescribing with clinical outcomes, including harms and benefits, but will not attempt to establish causality.

**Ethics and dissemination:**

The Health and Social Care Public Benefit and Privacy Policy Panel (HSC-PBPP) has granted ethical approval (ref. 2024-0239) following a Data Protection Impact Assessment, with data securely held and accessed in the National Safe Haven. The results will be published in international peer-reviewed journals and will be shared with clinicians.

**STRENGTHS AND LIMITATIONS OF THIS STUDY**

- Use of multiple national datasets, covering prescriptions as well as medicine administrations, enables robust capture of exposure to the medicines of interest.
- Operationalisation of both binary and time-dependent continuous exposure measures provides a nuanced assessment.
- Applying two methods, high-dose calculation (British National Formulary and chlorpromazine equivalents) allows comparison and validation of exposure definitions.
- Lack of electronic data for psychiatric inpatient settings restricts analyses, limiting the completeness of the cohort exposure.
- Potential challenges with missing or incomplete data across linked datasets may introduce bias, even with imputation strategies.

## Introduction

 Effective antipsychotic agents are a vital component of modern psychiatry, enabling the management of various mental health disorders such as schizophrenia, bipolar disorder and psychotic depression. UK audit data from the Prescribing Observatory for Mental Health indicate that over one-third of psychiatric inpatients are prescribed high-dose antipsychotics, and around 43% receive antipsychotic polypharmacy. Other hospital-based studies have reported high-dose prescribing rates of approximately 20%–25% among inpatients.^1 2^ Alongside this, evidence shows that antipsychotic use, even at standard therapeutic doses, carries a risk of serious adverse outcomes, including stroke, myocardial infarction, pneumonia, fractures and acute kidney injury, with risks appearing particularly elevated in vulnerable populations such as those with dementia, especially within the first week of treatment.^3^ However, in cases where patients with severe mental health conditions, such as treatment-resistant schizophrenia or other complex psychiatric disorders, do not respond to standard treatment, high-dose antipsychotic therapy may be necessary. This approach involves prescribing antipsychotics at doses higher than the maximum recommended licensed limits or using a combination of antipsychotic medicines. In our study, we define high-dose prescribing using the British National Formulary (BNF) criteria, where a high dose is considered as the use of a single antipsychotic above the recommended maximum daily dose, or the concurrent use of two or more antipsychotics whose combined percentage of the maximum dose exceeds 100%. While this can provide symptom relief for some, it carries significant risks, needing more frequent and intensive oversight due to their increased risk.^4 5^ Moreover, the limited evidence supporting the effectiveness of high-dose regimens compared with standard doses underscores the need for careful consideration, monitoring and individualised treatment planning.^6 7^

The use of high-dose antipsychotics has been observed among hospitalised patients in both psychiatric and non-psychiatric hospitals (where clinicians may not have specialised expertise in mental health treatment, raising concerns about the potential for inappropriate prescribing).^5 8^ High-dose therapy is associated with increased risks of adverse effects, including extrapyramidal symptoms, metabolic disturbances and cardiovascular issues.^9^,^22^

Effective prescribing practices are crucial to balancing the benefits of high-dose antipsychotic use against these risks. Antipsychotic stewardship, which involves monitoring drug usage and addressing factors contributing to overuse or misuse, is essential for promoting safe, evidence-based prescribing.^23^ By ensuring regular evaluation and careful adjustment of treatment plans, stewardship helps reduce the likelihood of adverse outcomes, particularly for vulnerable patients who may not have access to specialised psychiatric care.^4 5^

This study aims to evaluate the prevalence and impact of high-dose antipsychotic prescribing among hospitalised patients in acute care settings in Scotland, assessing both the benefits and potential harms on clinical outcomes. Research questions for this specific study include:

1. What is the prevalence of high-dose antipsychotic prescribing in various hospital settings, and what factors are associated with the use of high-dose antipsychotics?
2. What are the clinical profiles and characteristics of patients prescribed high-dose antipsychotics in hospital settings in Scotland?
3. What are the short-term impacts (outcomes that happen during or soon after the hospital stay, such as immediate side effects or changes in medication) and long-term impacts (outcomes that occur later, including readmissions, ongoing treatment or other health issues tracked through linked datasets) of high-dose antipsychotic use, and how do they compare to those associated with standard-dose regimens?
4. How can a multivariate model be developed using machine learning techniques that enhance decision-making effectiveness for high-dose antipsychotic prescribing in acute care settings, ultimately improving patient outcomes and safety?

This study design has several strengths and limitations that warrant consideration. By drawing on multiple national datasets (Hospital Electronic Prescribing and Medicines Administration (HEPMA), Scottish Morbidity Records (SMR) and Prescribing Information System (PIS)), we are able to robustly capture prescribing and administration data across acute hospital populations. The use of both binary and time-dependent continuous exposure measures allows a more nuanced characterisation of antipsychotic use, while applying two equivalence methods (BNF-defined doses and chlorpromazine equivalents) enables comparison and validation of exposure definitions, an approach well supported in the literature. At the same time, the absence of electronic data for psychiatric inpatient settings restricts analyses to acute hospital populations, limiting completeness and potential challenges with missing or incomplete data across linked datasets that may introduce bias even with imputation strategies. We also acknowledge that potential secular trends, such as changes in clinical guidelines or antipsychotic availability between 2019 and 2023, may have influenced prescribing patterns. Overall, these considerations highlight both the methodological strengths of our approach and the limitations that need to be acknowledged in interpreting the findings.

## Methods and analysis

### Research design

#### Data sources

NHS Scotland, part of the UK’s taxpayer-funded, devolved healthcare system, provides free healthcare to a population of 5.3 million across 14 diverse health boards, covering both urban and rural areas with varying levels of socioeconomic deprivation. The inpatient medicines data for this study will be sourced from the electronic HEPMA systems, which have been implemented across six of these 14 health boards in Scotland (online supplemental table 1). Also to support this study, we will use data from 12 linked national health datasets within NHS Scotland, which include prescribing records, hospital admissions, intensive care data and regional laboratory information. Together, these datasets provide a comprehensive view of patient demographics, clinical encounters and health outcomes across Scotland (table 1).

**Table 1:** Data source for cohort identification, study exposure, outcomes and covariates

Dataset	Data description	Use
Hospital Electronic Prescribing and Medicines Administration Scotland (HEPMA)	A digital platform used in hospitals to manage and record prescribing and administration of medications	Cohort identification, exposure, outcomes, covariates
Prescribing Information System (PIS)	A national database in Scotland that records and monitors prescriptions dispensed in the community, providing comprehensive data on outpatient medications	Exposure, outcomes, covariates
Scottish Morbidity Records 00 (SMR00)	A dataset in Scotland that captures outpatient activity information, including details on outpatient appointments, diagnoses and treatments across NHS Scotland, primarily in hospital and clinic settings	Outcomes, covariates
Scottish Morbidity Record 01 (SMR01)	A dataset in Scotland that records information on *acute hospital admissions*	Outcome, covariates
Scottish Morbidity Record 02 (SMR02)	A dataset in Scotland that captures information on *maternity hospital admissions*	Outcome, covariates
Scottish Morbidity Record 04 (SMR04)	Records information on **mental health inpatient and day-case admissions**	Covariates
Scottish Morbidity Record 06 (SMR06)	Records information on **cancer registrations**	Covariates
National Records of Scotland (NRS) Deaths	Records and compiles information on all deaths occurring in Scotland	Outcomes
Scottish Intensive Care Society Audit Group (SICSAG)	A dataset that collects detailed clinical information on patients admitted to intensive care units (ICUs) across Scotland	Outcomes
Scottish Renal Registry (SRR)	A dataset that collects and monitors clinical information related to patients with kidney disease in Scotland	Outcomes, covariates
General Practitioner (GP)	Refers to a family doctor or primary care physician who provides healthcare services such as diagnosis, treatment and referrals for patients in a community setting	Covariates
Scottish Care Information (SCI) Store Labs	A central repository for laboratory test results in Scotland	Covariates

#### Population and cohort

**Figure 1:**
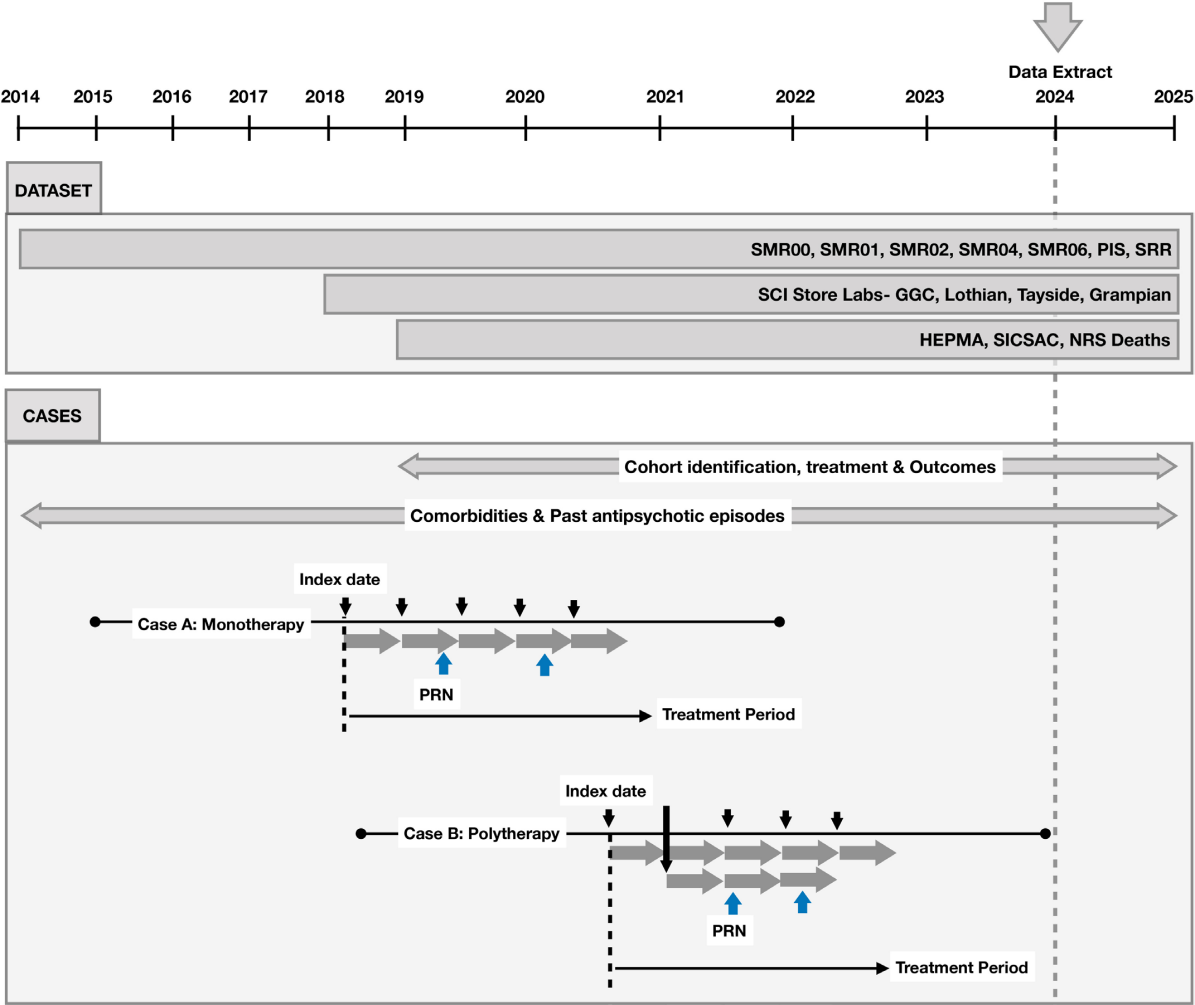
Timeline of data collection for each dataset, highlighting the identification of the cohort, treatment and comorbidities of patients. The index dates for both monotherapy and polytherapy cases are also illustrated. HEPMA, Hospital Electronic Prescribing and Medicines Administration Scotland; NRS, National Records of Scotland; PIS, Prescribing Information System; PRN, pro re nata; SCI, Scottish Care Information; SMR, Scottish Morbidity Record; SRR, Scottish Renal Registry.

The retrospective cohort study will include all hospitalised patients prescribed antipsychotics between January 2019 and December 2023 (figure 1). HEPMA, however, does not record prescribing indications, so it will include all hospitalised patients receiving antipsychotics. We will later use admission reasons and care specialty from SMR01 as proxies to identify psychiatric indications and focus part of the analysis on this subgroup. This will allow us to limit part of the analysis to patients likely to have psychiatric indications, providing a more focused assessment while maximising the utility of the available data. The first-ever antipsychotic prescription primarily applies to HEPMA, whereas we will also use the PIS database to identify prior antipsychotic prescribing. Specifically, we will apply a 1-year lookback period in PIS to assess whether patients had any previous antipsychotic prescriptions. Patients are required to have at least 1 year of prior data within SMR and PIS datasets before the index date (starting no earlier than January 2018 for a January 2019 cohort entry), and sufficient data to facilitate at least 1 year of follow-up post-index (latest cohort entry date is December 2022 depending on date of the latest data point available) to identify baseline characteristics and ascertain study outcomes, respectively.

#### Study exposure and comparison group

The maximum licensed dose of a drug includes the dosage range that has been demonstrated to give the best balance between the desired clinical effect and unwanted side effects.^4^,^6^ Exposure to high doses of antipsychotic medication refers to use of doses exceeding the recommended maximum dose for a single antipsychotic medicine as the licensed dose defined in the BNF.^4 24 25^ Additionally, concurrent prescribing/administration of more than one antipsychotic can lead to cumulative exposure to high doses. In such cases, the total daily cumulative dose is assessed through two methods:

1. By converting the dose of each drug into ‘chlorpromazine equivalents’ mg/day and adding these together.^26^ A cumulative dose of more than 1000 mg/day (ie, the maximum BNF daily dose for chlorpromazine) in chlorpromazine equivalents will be defined as a high dose.
2. By converting the dose of each drug into a percentage of the BNF maximum recommended licensed dose for that drug and adding these together.^4 25^ Cumulative dose of more than 100% will be defined as a high dose. For example, the BNF maximum dose of olanzapine is 20 mg; therefore, 10 mg of olanzapine constitutes a 50% antipsychotic dose.

**Figure 2:**
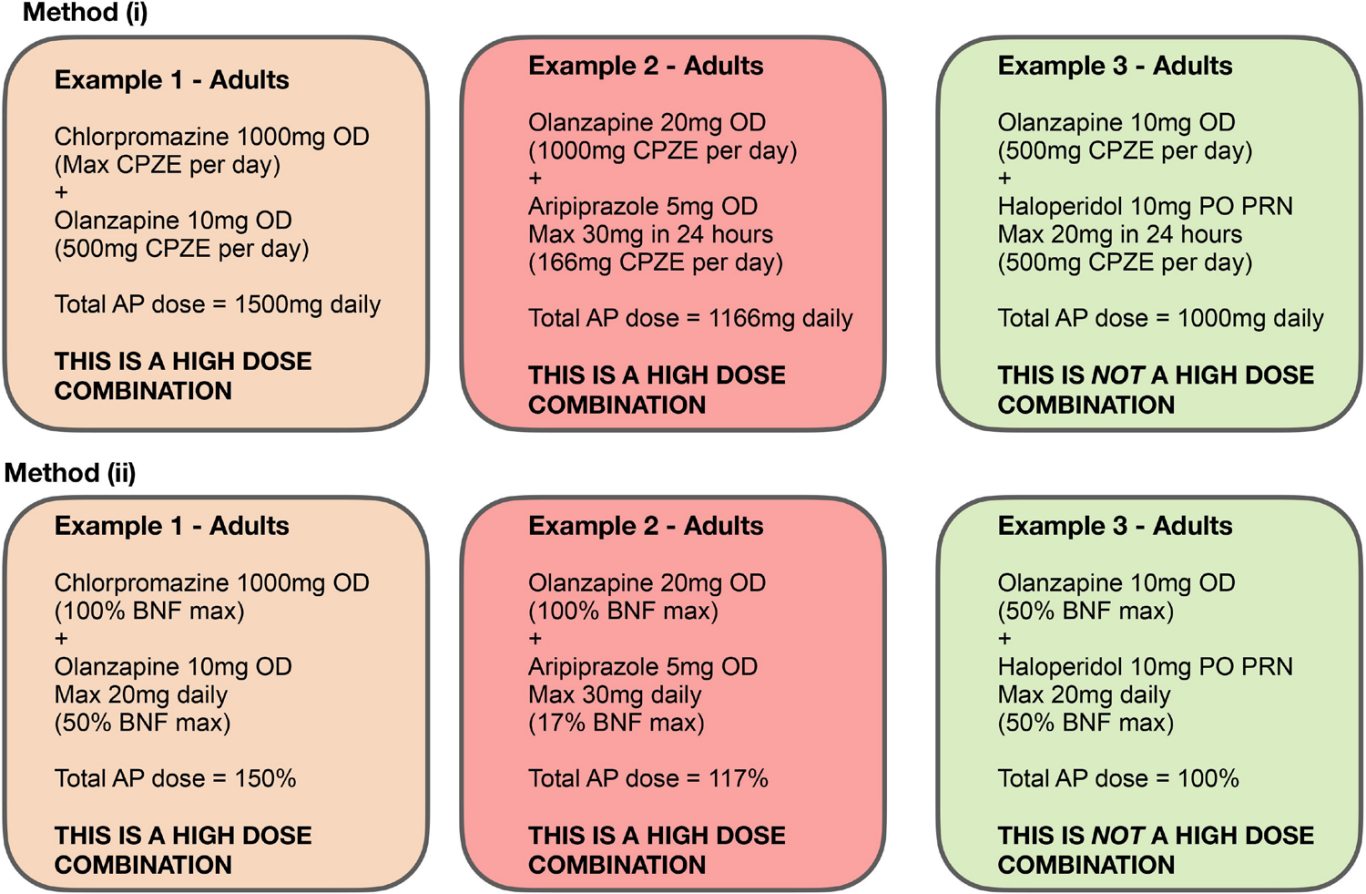
Examples of total antipsychotic (AP) daily dose calculation in cases of polytherapy, using two methods: (**i**) chlorpromazine equivalents (CPZE) and (ii) percentage of BNF (British National Formulary) maximum dose. OD, once a day; PRN, pro re nata.

Figure 2 shows three different examples of calculating total antipsychotic doses to identify patients with high-dose antipsychotics. Using both methods will allow us to compare results across different classification systems and assess whether findings are consistent regardless of the equivalence method applied. This dual approach strengthens the robustness of our analysis and reduces the risk of bias that may arise from relying on a single conversion scale. Furthermore, this approach is well documented in the literature,^27^ where both BNF-defined thresholds and chlorpromazine equivalents are frequently used in studies evaluating high-dose antipsychotic prescribing.

Patients will be followed from their first antipsychotic prescribing date (index date) in the hospital setting until death, study outcome or end of the study period, whichever comes first. The practice of prescribing high-dose antipsychotics could be either a new initiation with high dose or escalation of antipsychotic therapy (by increasing dose or adding another antipsychotic). Accordingly, different study design approaches will be considered, such as a new user design or a prevalent new user design, respectively, to account for such variation in practice.^28^ Missing data will be assessed across all linked datasets, and analyses will include all available observations. Where appropriate, multiple imputation will be applied for missing covariates, and sensitivity analyses will be conducted to evaluate the impact of missing data on study results.

**Figure 3:**
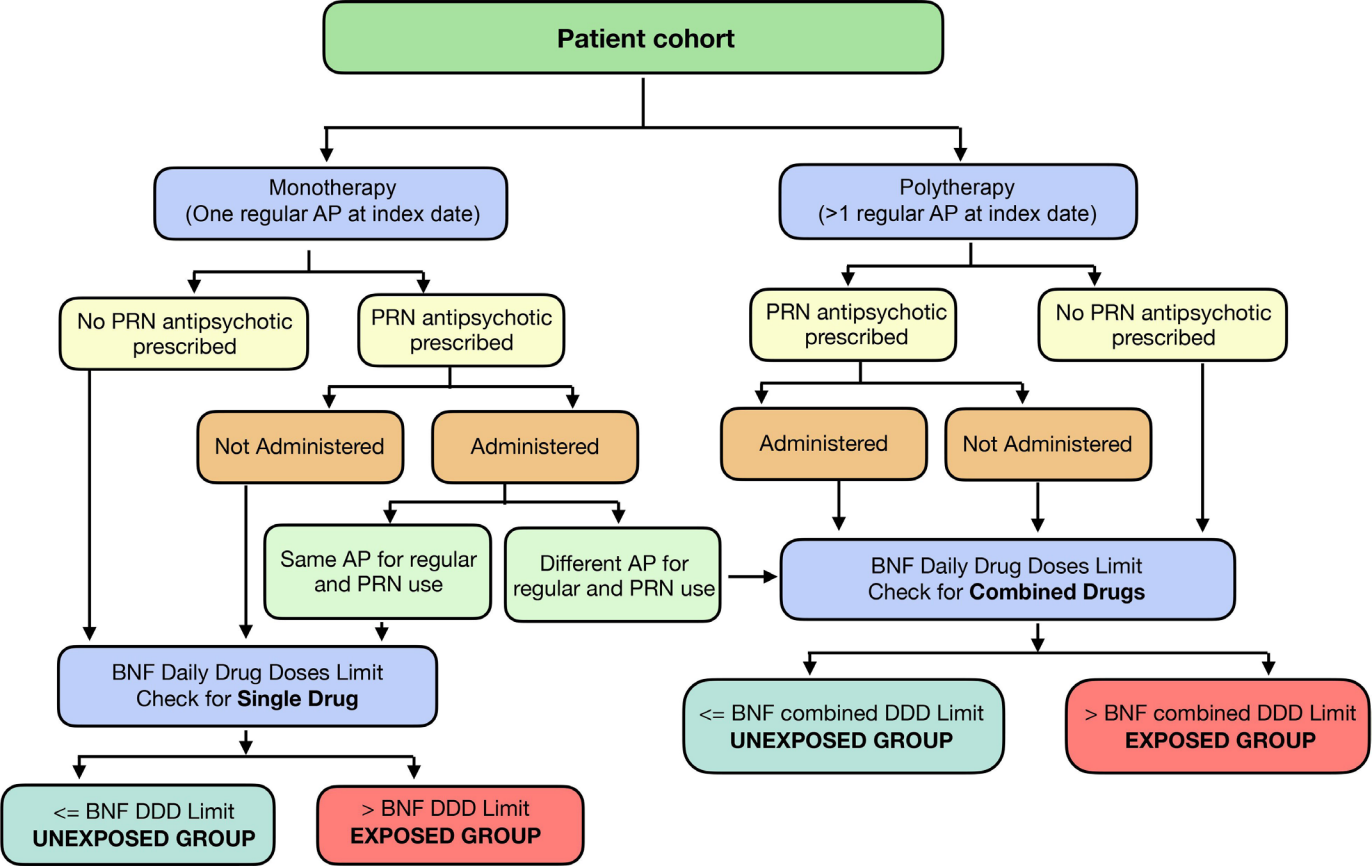
Assessment of high-dose antipsychotic (AP) use in monotherapy and polytherapy by categorising patients into exposed and unexposed groups based on PRN (pro re nata) medication administration. BNF, British National Formulary.

The drug exposure will be analysed in two separate ways. First, patients will be categorised into either the exposed or unexposed group (a binary measure). Patients will be considered exposed if they receive at least one administered dose of a high-dose antipsychotic, as captured in the HEPMA dataset. This ensures that all patients with any high-dose exposure are included in the analysis, while time-dependent measures will account for the duration and cumulative dosing. Unexposed patients will be defined as those who are prescribed antipsychotics but never exceed the high-dose threshold during the study period. For these patients, ‘time zero’ will be defined as the date of their first antipsychotic prescription, analogous to the index date for exposed patients, allowing comparable follow-up periods for outcome analyses. Second, we will consider the duration of actual exposure to high-dose antipsychotics based on administration data (a time-dependent continuous measure). For example, if a patient who is on chronic antipsychotic therapy and prescribed other antipsychotics as PRN (pro re nata) during a hospital admission which fulfils the definition of high dose antipsychotics, then this patient would be classed as an exposed patient if applying the first exposure definition even though the newly prescribed PRN was never administered; however, such a patient would not be classed as an exposed patient based on our second exposure measure (figure 3). Exposure to PRN antipsychotic prescriptions will be based solely on doses that were actually administered, with unadministered prescriptions excluded from both binary and time-dependent measures. While exposure data will be obtained from the HEPMA dataset, the exposure window will start at the index prescription date and continue until the earliest of the end of the study period, occurrence of a study outcome or patient death. This approach ensures that exposure measures accurately reflect actual patient intake rather than intended prescribing. Analyses will also account for potential biases from unadministered PRNs and incorporate appropriate methods to handle missing or incomplete data. Antipsychotics listed in the BNF (online supplemental table 2) will be included to capture a broad overview of prescribing practices. However, in the sensitivity analysis, drugs like levomepromazine and prochlorperazine will be excluded because they are frequently used for non-psychotic conditions and so their inclusion could falsely inflate the rates of high-dose prescribing.

To address potential immortal time bias, patients will enter the cohort at the date of their first antipsychotic prescription (index date), regardless of whether it is high-dose or standard-dose. High-dose exposure will be treated as a time-dependent variable, so that patients contribute person-time as unexposed until they first receive a high-dose antipsychotic, and as exposed thereafter. This approach ensures that follow-up time is appropriately allocated and prevents bias that could arise if patients were classified as exposed from cohort entry before receiving a high-dose prescription.

#### Key outcome measures

We will first quantify high-dose antipsychotic prescribing, then describe and profile the characteristics of the study cohort; the next step will be to assess the association between high-dose antipsychotic prescribing and clinical outcomes, including both risks and benefits. A multivariate model using machine learning techniques will be used to develop algorithms to identify/flag patients when high-dose antipsychotics are prescribed, as well as identify patients at risk of harm among those prescribed high-dose antipsychotics.

The clinical study outcomes will comprise both proxy measures of benefits (effectiveness) and harms (safety/proxy measures for adverse drug effects) (table 2). The effectiveness outcomes will be mainly related to the degree of psychiatric disease control. This will include frequency and length of psychiatric hospital admissions, mental health-related emergency department visits and change in adjunctive medication patterns such as anxiolytics, hypnotics and mood stabilisers (ie, expected to be reduced if the primary psychiatric condition is better managed). Use of high-dose antipsychotics can lead to severe adverse effects, including extrapyramidal symptoms (such as tremors and rigidity),^10 11^ metabolic disturbances (including weight gain, diabetes and hyperlipidaemia)^12^,^16^ and cardiovascular complications like QTc prolongation.^17^,^21^ Cognitive impairment, increased sedation and mortality are also frequent concerns.^22^

**Table 2:** List of the study outcome measures for high-dose antipsychotics, including both proxy measures of effectiveness (benefits) and safety (harms) with its relevant data sources

Study outcomes type	Clinical outcomes	Outcome measures	Operational definitions	Data source
Benefits	Psychiatric disease control	Frequency and length of psychiatric hospital admissions	ICD-10 codes	SMR01/04
		Emergency department (ED) visits	ICD-10 codes	SMR00/01
		Change in adjunctive medication patterns, such as anxiolytics, hypnotics, mood stabilisers	BNF codes for these medications	PIS, SMR04
Harms	Metabolic effects	Increased blood pressure	BP measurement and/or antihypertensive agents	SMR01, SRR, SCI Store
		High blood sugar	Blood glucose, Hb1Ac measurement and/or antidiabetics (insulin, OHA)	SMR01, SICSAG, SRR, SCI Store
		Abnormal cholesterol or TG	Lipid profile measurement and/or statins	SICSA G, SRR, SCI Store
		Diabetes	ICD-10 codes and/or antidiabetics (insulin, OHA)	SMR01/02, SCI Store Labs, SRR
		BMI/Weight Gain	BMI or increase in weight/waist circumference	GP, SMR01
	Cardiac effect	Arrhythmias, myocardial infarction, QTc prolongation	Diagnostic codes, procedure codes	SMR01/04, SICSAG
			Medication prescriptions	PIS
	Extrapyramidal symptoms	Dystonia, akathisia, Parkinsonism and tardive dyskinesia	Diagnosis and clinical notes	SMR01/04
			Anticholinergics (eg, benztropine, trihexyphenidyl, procyclidine): used to treat drug-induced Parkinsonism and dystonia	HEPMA, SMR04
			Dopamine agonists (eg, pramipexole, ropinirole): occasionally used for severe drug-induced Parkinsonism	HEPMA, SMR04
			Tetrabenazine: mostly used to treat movement disorder, target dyskinesia	HEPMA, SMR04
			Amantadine: can be used to treat drug-induced akathisia and tardive dyskinesia	HEPMA, SMR01/04, PIS
			Propranolol/benzodiazepine: generally used to reduce anxiety in case of akathisia	HEPMA, SMR01/04
	Cognitive		Diagnosis	SMR01/04
			Prescriptions for medications that may indicate treatment of cognitive symptoms or disorders associated with cognitive decline (eg, cholinesterase inhibitors like donepezil for dementia, stimulants for attention deficits)	SMR01/04/PIS
			ADHD-related drugs used mainly on younger population where the use of high-dose antipsychotics is more common	HEPMA, SMR01/04
			Dementia-related drug as a proxy in older population	HEPMA, SMR01/04
	Mortality	Psychiatric-related mortality All-cause mortality		NRS, SMR

HEPMA, Hospital Electronic Prescribing and Medicines Administration Scotland; ICD-10, International Classification of Disease, 10th Edition; NRS, National Records of Scotland; PIS, Prescribing Information System; SCI, Scottish Care Information; SICSAG, Scottish Intensive Care Society Audit Group; SMR, Scottish Morbidity Records; SRR, Scottish Renal Registry.

Outcomes such as weight gain or elevated blood pressure may result from factors other than high-dose antipsychotics, particularly in hospitalised patients. To address this, weight gain will be defined as an increase of ≥5% from baseline, and high blood pressure will require repeated measurements above standard clinical thresholds. Where appropriate, composite measures will be used to capture clinically meaningful changes and reduce misclassification, ensuring that outcome definitions are clear, consistent and clinically relevant. We will adjust for potential confounding factors, including critical illness, comorbidities and other relevant variables, to strengthen the validity of the independent association between the exposure and study outcomes. However, we acknowledge that, given the observational nature of the study design, the influence of unmeasured confounders cannot be fully excluded. Since we are looking at several outcomes, there is a chance of false positives. To handle this, we will clearly separate primary and secondary outcomes. For secondary outcomes, p values will be adjusted using the false discovery rate method, while exploratory analyses will be interpreted cautiously, emphasising effect sizes and CIs rather than statistical significance alone.

#### Study covariates and confounders

Patient characteristics (eg, age, gender, ethnicity, Scottish index of multiple deprivation (SIMD)) and other potentially relevant covariates, either related to the choice of treatment or the observed treatment outcomes, will be summarised at baseline, that is, as applicable at the index date.^6 7^ Other drugs from community prescribing (PIS) will be evaluated for both monotherapy and polypharmacy (antipsychotics and other drugs) in the 12 months prior to the index date, while comorbidities will be assessed using the Charlson Comorbidity Index over the 5 years leading up to the index date.^29^

In addition to what is available in HEPMA, diagnostic information (diabetes diagnoses, previous psychiatric care and comorbidities) will be obtained from SMR00/01/04/06 and the Scottish Care Information store; and data relating to previous, concomitant and/or referral reasons and diagnosis will be obtained from SMR00/01/04 . Detailed lists of all study outcomes, their definitions and data sources can be found in table 2. We acknowledge that some outcome definitions in table 2 may be subject to limitations such as misclassification, reliance on proxies and bias, and these will be examined, particularly when certain medications are used as proxies for specific outcomes.

### Data analysis

The analysis of linked, pseudonymised datasets will commence with descriptive analyses to characterise the cohort and assess the prevalence of high-dose antipsychotic prescribing, followed by statistical analyses aimed at addressing the stated research questions. All data preparation, data analyses and production of data visualisations will be undertaken in R, Python and potentially other software as required.

#### Formal statistical analysis

To understand the clinical profiles and characteristics of patients prescribed high-dose antipsychotics in Scottish hospitals, we will employ descriptive statistics. This will involve summarising key demographic and clinical variables, such as age, gender, SIMD, primary diagnosis, comorbidities and mental health treatment history. We will explore prevalence and associations through demographic and hospital size adjustments, regression models (Cox/LASSO regression) and χ^2^ tests to highlight trends in high dose prescribing among various categories.^30^

For a contemporary analysis of high-dose antipsychotic prescribing and related outcomes, a range of advanced statistical methods will be applied to gain deeper insights. Kaplan-Meier survival curves and Cox proportional HRs will be used to evaluate time-to-event analysis, such as metabolic syndrome or mortality.^31^ To address potential overfitting, bootstrap validation (with at least 1000 bootstraps) will be performed on each imputed dataset, allowing us to quantify optimism, estimate shrinkage factors and adjust regression coefficients.

Prediction models to identify risks and benefits associated with the high-dose antipsychotics will be employed to forecast individual patient responses to specific medications, while adjusting for confounding variables through applying various advanced techniques such as propensity score matching or instrumental variable analysis.^32^,^34^ Additionally, in an effort to account for unmeasured confounders, sensitivity analyses will be performed. Where outcomes rely on proxy indicators (eg, medication prescribing for extrapyramidal symptoms or cognitive decline), we will conduct sensitivity analyses in subsets with more detailed clinical information, compare findings across multiple proxy definitions and discuss potential misclassification bias in the interpretation of results. Furthermore, machine learning algorithms, including cross-validation and random forest classification, will be employed to enhance predictive accuracy and model generalisability.^35^ Random forest models will serve as the primary method, with gradient boosting (XGBoost, LightGBM) and regularised regression (LASSO, elastic net) included for comparison. Model performance will be assessed using area under the receiver operating characteristic, calibration metrics (Brier score, calibration plots) and interpretability through SHapley Additive exPlanations values and feature importance. To address potential confounding when comparing dose groups, propensity score matching and weighting methods will also be applied.

### Patient and public involvement

Patient and public involvement (PPI) was not included in the design or conduct of this research. However, dissemination of outcomes to the public may include newspaper articles, newsletters and lay summaries on the Health Data Research UK (HDR UK) website; established HDR UK processes (including PPIE) will be used to facilitate wide dissemination. In future iterations, PPI groups will be involved in developing dissemination materials, including lay summaries, to improve accessibility and relevance of findings.

## Ethics and dissemination

The Health and Social Care Public Benefit and Privacy Policy Panel (HSC-PBPP) has provided ethics approval for this research study (ref. 2024-0239) following completion of Data Protection Impact Assessment form. The study data are protected under generic ethical approval, as the data will be held and accessed within the National Safe Haven—a secure, trusted research environment, unconsented, de-identified data derived from electronic health records collected as part of patients’ routine healthcare. Findings will be disseminated through peer-reviewed journals, conferences, policy briefs and public outreach to maximise impact and influence mental health practices and policy. Collaborative sharing with partners will further enhance the research’s contribution to improving mental health outcomes.

## Supplementary material

10.1136/bmjopen-2025-098927 online supplemental file 1
