# Supplementary material for: Study protocol for optimising antipsychotic prescribing among hospitalised patients in the acute care setting in Scotland: a national retrospective cohort study
Source: BMJ Open. 2025 Dec 10;15(12):e098927. doi: 10.1136/bmjopen-2025-098927 (PMC12699559; doi:10.1136/bmjopen-2025-098927)
Supplement: online supplemental file 1 [file bmjopen-15-12-s001.pdf]

A study protocol for optimising antipsychotic prescribing among hospitalised patients in the acute care setting in Scotland: A national retrospective cohort study

Cosmika Goswami[1*], Tanja Mueller[1], Alexa Wall[2a], Chris F Johnson[3], David Grosset[2b], Marion Bennie[1], Amanj Kurdi[1,4,5.]

[1] Strathclyde University of Pharmacy and Biomedical Sciences, University of Strathclyde, UK

[2a] Pharmacy Services, National Health Services Lothian, UK

[2b] Psychiatry, National Health Services Lothian, UK

[3] Pharmacy Services, National Health Services Greater Glasgow & Clyde, UK

[4] College of Pharmacy, Al-Kitab University, Kirkuk 36015, Iraq

[5] Department of Public Health Pharmacy and Management, School of Pharmacy, Sefako Makgatho Health Sciences University, Pretoria, South Africa

**Corresponding author: Dr. Cosmika Goswami; cosmika.goswami@strath.ac.uk*

SUPPLEMENTAL MATERIAL

Supplementary Table 1: NHS Scotland National HEPMA Dataset Sources by Health Board, Population Coverage, and Available Years.

Health Board	% of Scottish Population[1]	Available
Ayrshire & Arran	6.7	2019
Dumfries & Galloway	2.7	2019
Forth Valley	5.6	2019
Grampian	10.7	2024
Greater Glasgow & Clyde	21.6	mid–2021[2]
Highland	5.9	2024

Lanarkshire	12.3	2019
Lothian	16.6	mid–2020[2]
Orkney	0.4	2023
Shetland	0.4	2023
Tayside	7.6	2023
Western Isles	0.5	2023
**Total Population Covered**	**90.6%**	

[1] Source for population data: National Records of Scotland (2022)

[2] GGC & Lothian Health Boards have staggered start dates across different hospitals/wards

Supplementary Table 2 Antipsychotics maximum Defined Daily Dosage (DDD) by BNF4.2

Approved Drug Name	BNF maximum defined daily doses	Administration Route
**Amisulpride[2]**	1200mg	Oral
**Aripiprazole[2]**	30mg	Oral & Parenteral
**Asenapine**	20mg	Oral
**Benperidol[1]**	1.5mg	Oral
**Cariprazine**	6mg	Oral
**Chlorpromazine [1]**	1000mg	Oral
**Clozapine[2]**	900mg	Oral
**Flupentixol[1]**	18mg	Oral
**Haloperidol[1]**	20mg	Oral

**Levomepromazine**[1]	1000mg	Oral
**Lurasidone**	148mg	Oral
**Olanzapine**[2]	20mg	Oral & Parenteral
**Paliperidone**[2]	12mg	Oral
**Pericyazine**[1]	300mg	Oral
**Perphenazine**[1]	24mg	Oral
**Pimozide**[1]	20mg	Oral
**Promazine**[1]	800mg	Oral
**Quetiapine**[2]	750mg	Oral
**Risperidone**[2]	16mg	Oral
**Sulpiride**[1]	2400mg	Oral
**Trifluoperazine**[1]	50mg	Oral
**Zuclopenthixol**[1]	150mg	Oral

[1] Typical drug [2] Atypical drug
