# Supplementary material for: Study protocol for optimising antipsychotic prescribing among hospitalised patients in the acute care setting in Scotland: a national retrospective cohort study
Source: BMJ Open. 2025 Dec 10;15(12):e098927. doi: 10.1136/bmjopen-2025-098927 (PMC12699559; doi:10.1136/bmjopen-2025-098927)
Supplement: Supplementary file 2 [file bmjopen-2025-098927.reviewer_comments.pdf]

PEER REVIEW HISTORY

BMJ Open publishes all reviews undertaken for accepted manuscripts. Reviewers are asked to complete a checklist review form and are provided with free text boxes to elaborate on their assessment. These free text comments are reproduced below.

ARTICLE DETAILS

Title (Provisional)

A study protocol for optimising antipsychotic prescribing among hospitalised patients in the acute care setting in Scotland: A national retrospective cohort study

Authors

Goswami, Cosmika; Mueller, Tanja; Wall, Alexa; Johnson, Chris F; Grosset, David; Bennie, Marion; Kurdi, Amanj

---

VERSION 1 - REVIEW

**Reviewer**	**1**
**Name**	**Elhusein, Bushra**
**Affiliation**	**Hamad Medical Corporation, Psychiatry**
**Date**	**19-May-2025**
**COI**	**None**

Thank you for the opportunity to review this study protocol examining antipsychotic prescribing among hospitalized patients in Scotland. Overall, it seems very well written and promising; please find my comments below.

The paper refers to “polytherapy” as one of the keywords; however, in pharmacological and clinical contexts, especially in psychiatry, polypharmacy is more appropriate when referring to the concurrent use of multiple medications, and polytherapy may lead to ambiguity, as it can refer to other treatment modalities.

The authors stated " first-ever antipsychotic prescription,” which is reasonable as the index date, but additional details are needed on how this will be determined retrospectively across the HEPMA, SMR, and PIS databases. Is there a lookback period to ensure that the index prescription truly represents the first exposure?

Page 5, lines 6-11: It’s advisable to revise this line to emphasize that antipsychotic use, regardless of dosage, necessitates regular monitoring to prevent adverse effects. Due to the increased risk, it’s important to note that patients on higher doses will need more frequent monitoring. Saying they require regular monitoring might imply that those on standard therapeutic doses do not need oversight.

Is the study looking for non-specialist prescribers or also psychiatrists?

Page 6. Line 13. It will be good considering what you mean by short-term and long-term impact.

The authors provide a good approach to drug exposure classification (binary and time-dependent continuous measures); however, the example provided regarding prn prescriptions that were never administered raises some methodological concerns. It would strengthen the manuscript to clearly define how exposure windows will be operationalized in both measures and also discuss how potential exposure, e.g prescribed but not administered PRNs, will be handled analytically.

**Reviewer**	**2**
**Name**	**Lewis, Kimberley**
**Affiliation**	**McMaster University, Department of Medicine**
**Date**	**04-Jun-2025**
**COI**	**None**

Thank you for the opportunity to review the manuscript by Goswami and colleagues. They have written a protocol examining dosing of antipsychotics in patients admitted to an acute care setting.

Overall, the protocol was written well. I have a inquiries

1-I think the authors need to explicitly state what they consider to be "high-dose antipsychotics". This would be great information to put in a table (either in the manuscript or in a supplement)

2-I don't understand the rationale for converting all antipsychotics to chlorpromazine equivalents.

3-A description of "short term" and "long term" impact needs to be further described.

4-I think you need to specify your patient population-is this psychiatric and acute-medical/surgical admissions (just needs to be better defined) as your primary outcome seems to be related to psychiatric conditions.

5-There needs to be a discussion on why this is relevant, the strengths and limitations of the design, and how this builds off prior work

---

**Reviewer**	**3**
**Name**	**Jaworska, Natalia**

Affiliation University of Calgary, Critical Care Medicine

Date 18-Jun-2025

COI None

Thank you for the opportunity to review your work.

The authors present a study protocol for a national retrospective cohort study of evaluating antipsychotic prescribing among hospitalized patient in acute care settings in Scotland. The authors' aim is to understand current prescribing practices and assess the impact of high-dose antipsychotic prescribing on clinical outcomes among hospitalized patients. The study outcomes will be used to develop an integrated computerized clinical decision support tool.

I commend the authors on the substantial work put forth to complete this study protocol. This is an important topic as inappropriate prescribing of high-dose antipsychotics exposes patients to significant risk of adverse events. Appropriate dosing and prescribing practices are essential to minimize the risks associated with antipsychotic use. The manuscript is overall clear and well-written. I provide the following suggestions for strengthening the manuscript.

1. High-dose antipsychotic prescribing frequently arises as a result of difficult to manage psychiatric disease. Antipsychotic stewardship is certainly a potentially important mechanism to minimize adverse outcomes. What proportion of patients with psychiatric disease receive high-dose antipsychotics? What proportion of patients on high-dose antipsychotics have adverse events in other settings? Providing this information in the background would provide stronger rationale for this study.

2. The indication for prescribing antipsychotics varies across different patient cohorts. The rationale for including all hospitalized patients prescribed antipsychotics is unclear. Most patients receiving high-dose antipsychotics are for indications of psychiatric diagnoses and prescribing of high-dose antipsychotics is usually guided with psychiatric consultation and subspecialists. What is the rationale for including all admitted patients rather than patients with psychiatric diagnoses in the cohort definition?

3. Table 1 is clear and a helpful visualization as to the sources of data for this cohort study.

4. The challenges with data linkage across multiple databases includes challenges with missing data. How will missingness be managed? Will those with missing data be excluded or will other methodologies such as multiple imputations be used?

5. The exposure definition for high-dose antipsychotic is complex but reasonably well defined by the authors. What remains unclear is how many doses of high-dose antipsychotics will be needed to be defined as being exposed. Will one dose of the defined high-dose antipsychotic be sufficient to count as exposure? How many received doses is sufficient to define exposure? How will unexposed patients be defined and how will their "time zero" be defined given they are unexposed?

6. Some patients may enter the cohort and become exposed to high-dose antipsychotics during the study period potentially generating immortal time bias. How will immortal time bias be managed in this study? Will patients enter the cohort after first antipsychotic exposure or first high-dose antipsychotic exposure?

7. Some outcomes will be extracted from clinic notes. How will this be done? Is there plan for a natural language processing model to acquire this data? Reviewing individual charts/clinic notes for such a sizable cohort would not seem feasible.

8. There are multiple outcomes of interest for this study which certainly increases the risk of testing error from multiple comparisons. Is there risk for Type 1 error and if so, how will this be managed?

9. Some adverse events take longer than others to occur and can be difficult to correlate (e.g., diabetes, weight gain) with the exposure. Particularly when evaluating a patient cohort with all hospitalized patients, there may be many reasons other than high-dose antipsychotics responsible for weight gain. For example, critically ill patients may be exposed to high-dose antipsychotics and gain weight related to critical illness rather than high-dose antipsychotics. Further, how will outcome variables such as weight gain or increased blood pressure be defined? Will it be defined as a single higher weight or blood pressure measurement or will to be a defined as a composite variable?

VERSION 1 - AUTHOR RESPONSE

Reviewer: 1
Dr. Bushra Elhusein, Hamad Medical Corporation
Comments to the Author:
Thank you for the opportunity to review this study protocol examining antipsychotic prescribing among hospitalized patients in Scotland. Overall, it seems very well written and promising; please find my comments below.

1.1 The paper refers to “polytherapy” as one of the keywords; however, in pharmacological and clinical contexts, especially in psychiatry, polypharmacy is more appropriate when referring to the concurrent use of multiple medications, and polytherapy may lead to ambiguity, as it can refer to other treatment modalities.

Answer: In our manuscript, the term polytherapy is intentionally used to describe the concurrent use of more than one antipsychotic medication. We agree this is distinct from polypharmacy, which is more commonly used to describe the concurrent use of multiple medications across different therapeutic classes. We believe polytherapy is therefore more precise in the context of our study on antipsychotic prescribing patterns. However, we agree that in the methods section (study covariates and confounders) on page 15, where the discussion extends beyond antipsychotics to include other drugs, the use of the

term polypharmacy is more appropriate, and we have revised that accordingly. (Changes added to the main document, page 15, line 329)

1.2 The authors stated " first-ever antipsychotic prescription," which is reasonable as the index date, but additional details are needed on how this will be determined retrospectively across the HEPMA, SMR, and PIS databases. Is there a lookback period to ensure that the index prescription truly represents the first exposure?

Answer: The definition of "first-ever antipsychotic prescription" primarily applies to HEPMA, which serves as our main source for exposure. To strengthen our approach, we will also use the PIS database to identify prior antipsychotic prescribing. Specifically, we will apply a one-year lookback period in PIS to assess whether patients had any previous antipsychotic prescriptions. This will allow us to more accurately establish new incident users and ensure that the index prescription truly represents first exposure within the scope of the available data. (Changes added to the main document, page 8, lines 184-188)

1.3 Page 5, lines 6-11: It's advisable to revise this line to emphasize that antipsychotic use, regardless of dosage, necessitates regular monitoring to prevent adverse effects. Due to the increased risk, it's important to note that patients on higher doses will need more frequent monitoring. Saying they require regular monitoring might imply that those on standard therapeutic doses do not need oversight.

Answer: The text had been made clearer and highlighted that everyone on antipsychotics needs regular monitoring to catch any side effects. Changed in the manuscript - "While this can provide symptom relief for some, it carries significant risks, needing more frequent and intensive oversight due to their increased risk [1, 2]". (Changes added to the main document, page 4, lines 105-106)

1.4 Is the study looking for non-specialist prescribers or also psychiatrists?

Answer: The study will include prescribing by both psychiatrists and non-specialist prescribers in acute care of hospital settings, as our aim is to capture the full scope of antipsychotic prescribing practices across clinical settings. (already mentioned in Introduction section, page 4, lines 109-111, so no more changes made to the main document)

1.5 Page 6. Line 13. It will be good considering what you mean by short-term and long-term impact.

Answer: By short-term impact, we mean outcomes that happen during or soon after the hospital stay, such as immediate side effects or changes in medication. Long-term impact refers to outcomes that occur later, including readmissions, ongoing treatment, or other health issues tracked through linked datasets. (Changes added to the main document, page 5, lines 136-139)

1.6 The authors provide a good approach to drug exposure classification (binary and time-dependent continuous measures); however, the example provided regarding prn prescriptions that were never administered raises some methodological concerns. It would strengthen the manuscript to clearly define how exposure windows will be operationalized in both measures and also discuss how potential exposure, e.g., prescribed but not administered PRNs, will be handled analytically.

Answer: Exposure to PRN (pro re nata) antipsychotic prescriptions will be based solely on doses that were actually administered, with unadministered prescriptions excluded from both binary and time-dependent measures. Exposure data will be obtained from HEPMA dataset, which provide detailed records of prescribed and administered medications. The exposure window will start at the index prescription date and continue until the earliest of the end of the study period, occurrence of a study outcome, or patient death. This approach ensures that exposure measures accurately reflect actual patient intake rather than intended prescribing. Analyses will also account for potential biases from unadministered PRNs and incorporate appropriate methods to handle missing or incomplete data. (Changes added to the main document, page 10, lines 255-264)

Reviewer: 2

Dr. Kimberley Lewis, McMaster University

Comments to the Author:

Thank you for the opportunity to review the manuscript by Goswami and colleagues. They have written a protocol examining dosing of antipsychotics in patients admitted to an acute care setting.

Overall, the protocol was written well. I have a inquiries

2.1-I think the authors need to explicitly state what they consider to be "high-dose antipsychotics". This would be great information to put in a table (either in the manuscript or in a supplement)

Answer: In our study, we define high-dose prescribing using the British National Formulary (BNF) criteria, where a high dose is considered as the use of a single antipsychotic above the recommended maximum daily dose, or the concurrent use of two or more antipsychotics whose combined percentage of the maximum dose exceeds 100%. (Changes added to the main document, page 4, lines 100-103)

2.2-I don't understand the rationale for converting all antipsychotics to chlorpromazine equivalents.

Answer: We plan to use two approaches for defining high-dose: BNF-defined dose equivalence and chlorpromazine equivalents. Using both methods will allow us to compare results across different classification systems and assess whether findings are consistent regardless of the equivalence method applied. This dual approach strengthens the robustness of our analysis and reduces the risk of bias that may arise from relying on a single conversion scale. Furthermore, this approach is well documented in the literature, Yorston and Pinney (2000), where both BNF-defined thresholds and chlorpromazine equivalents are frequently used in studies evaluating high-dose antipsychotic prescribing.

Reference:
Yorston G, Pinney A. *Chlorpromazine equivalents and percentage of British National Formulary maximum recommended dose in patients receiving high-dose antipsychotics.* Psychiatric Bulletin. 2000;24:130–132.

(Changes added to the main document, page 9, lines 218-224)

2.3-A description of "short term" and "long term" impact needs to be further described.

Answer: By short-term impact, we mean outcomes that happen during or soon after the hospital stay, such as immediate side effects or changes in medication. Long-term impact refers to outcomes that occur later, including readmissions, ongoing treatment, or other health issues tracked through linked datasets. (Changes added to the main document, page 5, lines 136-139)

2.4-I think you need to specify your patient population-is this psychiatric and acute-medical/surgical admissions (just needs to be better defined) as your primary outcome seems to be related to psychiatric conditions.

Answer: We included all hospitalised patients in acute care settings who were prescribed antipsychotics, across both psychiatric and non-psychiatric hospitals. The use of available hospital acute care datasets allows us to capture real-world antipsychotic prescribing in these

settings. While we recognise that most high-dose prescriptions are typically guided by psychiatric consultation, a key limitation is that dedicated psychiatric admissions are not captured due to data privacy restrictions. Importantly, the outcomes of interest, although psychiatric in nature, are not limited to patients admitted solely to psychiatric hospitals. Including both psychiatric and non-psychiatric hospital settings therefore enables us to capture the full spectrum of relevant cases. (No change made to the main document)

2.5-There needs to be a discussion on why this is relevant, the strengths and limitations of the design, and how this builds off prior work

Answer: This study design has several strengths and limitations that warrant consideration. By drawing on multiple national datasets (HEPMA, SMR, and PIS), we are able to robustly capture prescribing and administration data across acute hospital populations. The use of both binary and time-dependent continuous exposure measures allows a more nuanced characterisation of antipsychotic use, while applying two equivalence methods (BNF-defined doses and chlorpromazine equivalents) enables comparison and validation of exposure definitions, an approach well supported in the literature. At the same time, the absence of electronic data for psychiatric inpatient settings restricts analyses to acute hospital populations, limiting completeness, and potential challenges with missing or incomplete data across linked datasets may introduce bias even with imputation strategies. Overall, these considerations highlight both the methodological strengths of our approach and the limitations that need to be acknowledged in interpreting the findings. (Changes added to the main document, page 5, lines 145-157)

Reviewer: 3

Dr. Natalia Jaworska, University of Calgary

Comments to the Author:

Thank you for the opportunity to review your work.

The authors present a study protocol for a national retrospective cohort study of evaluating antipsychotic prescribing among hospitalized patient in acute care settings in Scotland. The authors' aim is to understand current prescribing practices and assess the impact of high-dose antipsychotic prescribing on clinical outcomes among hospitalized patients. The study outcomes will be used to develop an integrated computerized clinical decision support tool.

I commend the authors on the substantial work put forth to complete this study protocol. This is an important topic as inappropriate prescribing of high-dose antipsychotics exposes patients to significant risk of adverse events. Appropriate dosing and prescribing practices are essential to minimize the risks associated with antipsychotic use. The manuscript is overall clear and well-written. I provide the following suggestions for strengthening the manuscript.

3.1. High-dose antipsychotic prescribing frequently arises as a result of difficult to manage psychiatric disease. Antipsychotic stewardship is certainly a potentially important mechanism to minimize adverse outcomes. What proportion of patients with psychiatric disease receive high-dose antipsychotics? What proportion of patients on high-dose antipsychotics have adverse events in other settings? Providing this information in the background would provide stronger rationale for this study.

Answer: High-dose antipsychotic prescribing is relatively common in psychiatric practice and is often associated with complex or treatment-resistant illness. UK audit data from Prescribing Observatory for Mental Health (POMH-UK) indicate that over one-third of psychiatric inpatients are prescribed high-dose antipsychotics, and around 43% receive antipsychotic polypharmacy. Other hospital-based studies have reported high-dose prescribing rates of approximately 20-25% among inpatients [1, 2]. Alongside this, evidence shows that antipsychotic use, even at standard therapeutic doses, carries a risk of serious adverse outcomes, including stroke, myocardial infarction, pneumonia, fractures, and acute kidney injury, with risks appearing particularly elevated in vulnerable populations such as those with dementia, especially within the first week of treatment [3]. Taken together, these data highlight both the prevalence of high-dose prescribing and the significant potential for harm, thereby providing a strong rationale for our study and for exploring interventions that can improve antipsychotic stewardship.

References:

1. Luthra S, Duggan L, Agrawal A, Kaur G, Luthra N. Prevalence of High-dose Antipsychotic Prescribing in Schizophrenia: A Clinical Audit in a Regional Queensland Mental Health Service. Int J Appl Basic Med Res. 2023 Apr-Jun;13(2):70-76. doi: 10.4103/ijabmr.ijabmr_504_22.
2. Mace, Shubhra & Taylor, David. Reducing the rates of prescribing high-dose antipsychotics and polypharmacy on psychiatric inpatient and intensive care units: results of a 6-year quality improvement programme. Therapeutic advances in psychopharmacology 2015. 5. 4-12. 10.1177/2045125314558054.
3. Mok P L H, Carr M J, Guthrie B, Morales D R, Sheikh A, Elliott R A et al. Multiple adverse outcomes associated with antipsychotic use in people with dementia: population based matched cohort study BMJ 2024; 385: e076268 doi:10.1136/bmj-2023-076268

(Changes added to the main document, page 3, lines 70-96)

3.2. The indication for prescribing antipsychotics varies across different patient cohorts. The rationale for including all hospitalized patients prescribed antipsychotics is unclear. Most patients receiving high-dose antipsychotics are for indications of psychiatric diagnoses and prescribing of high-dose antipsychotics is usually guided with psychiatric consultation and subspecialists. What is the rationale for including all admitted patients rather than patients with psychiatric diagnoses in the cohort definition?

Answer: We completely agree that ideally the cohort should include only patients prescribed antipsychotics for psychiatric indications. HEPMA, however, does not record prescribing indications, so will include all hospitalized patients receiving antipsychotics. We will later use admission reasons and care specialty from SMR01 as proxies to identify psychiatric indications and focus part of the analysis on this subgroup. This will allow us to limit part of the analysis to patients likely to have psychiatric indications, providing a more focused assessment while maximising the utility of the available data. (Changes added to the main document, Page 8, lines 179-188)

3.3. Table 1 is clear and a helpful visualization as to the sources of data for this cohort study.

Thank you

3.4. The challenges with data linkage across multiple databases includes challenges with missing data. How will missingness be managed? Will those with missing data be excluded or will other methodologies such as multiple imputations be used?

Answer: Missing data will be assessed across all linked datasets, and analyses will include all available observations. Where appropriate, multiple imputation will be applied for missing covariates, and sensitivity analyses will be conducted to evaluate the impact of missing data on study results. This information is included in the methods section. (Changes added to the main document, page 10, lines 234-237)

3.5. The exposure definition for high-dose antipsychotic is complex but reasonably well defined by the authors. What remains unclear is how many doses of high-dose antipsychotics will be needed to be defined as being exposed. Will one dose of the defined high-dose antipsychotic be sufficient to count as exposure? How many received doses is sufficient to define exposure? How will unexposed patients be defined and how will their “time zero” be defined given they are unexposed?

Answer: For this study, patients will be considered exposed if they receive at least one administered dose of a high-dose antipsychotic, as captured in the HEPMA dataset. This ensures that all patients with any high-dose exposure are included in the analysis, while time-dependent measures will account for the duration and cumulative dosing. Unexposed patients will be defined as those who are prescribed antipsychotics but never exceed the high-dose threshold during the study period. For these patients, "time zero" will be defined as the date of their first antipsychotic prescription, analogous to the index date for exposed patients, allowing comparable follow-up periods for outcome analyses. Sensitivity analyses will also explore alternative exposure definitions, such as requiring multiple high-dose administrations, to assess the robustness of the findings. (Changes added to the main document, page 10, lines 240-248)

3.6. Some patients may enter the cohort and become exposed to high-dose antipsychotics during the study period potentially generating immortal time bias. How will immortal time bias be managed in this study? Will patients enter the cohort after first antipsychotic exposure or first high-dose antipsychotic exposure?

Answer: To address potential immortal time bias, patients will enter the cohort at the date of their first antipsychotic prescription (index date), regardless of whether it is high-dose or standard dose. High-dose exposure will be treated as a time-dependent variable, so that patients contribute person-time as unexposed until they first receive a high-dose antipsychotic, and as exposed thereafter. This approach ensures that follow-up time is appropriately allocated and prevents bias that could arise if patients were classified as exposed from cohort entry before receiving a high-dose prescription. (Changes added to the main document, page 11, lines 270-276)

3.7. Some outcomes will be extracted from clinic notes. How will this be done? Is there plan for a natural language processing model to acquire this data? Reviewing individual charts/clinic notes for such a sizable cohort would not seem feasible.

Answer: Outcomes derived from clinical notes will not be included in this study, as access to such records is not available. Therefore, neither manual chart review nor natural language processing approaches will be employed to extract data from clinical notes for this cohort. (No change made to the main document)

3.8. There are multiple outcomes of interest for this study which certainly increases the risk of testing error from multiple comparisons. Is there risk for Type 1 error and if so, how will this be managed?

Answer: Since we are looking at several outcomes, there's a chance of false positives. To handle this, we will clearly separate primary and secondary outcomes. For secondary outcomes, p-values will be adjusted using the false discovery rate (FDR) method, while exploratory analyses will be interpreted cautiously, emphasising effect sizes and confidence intervals rather than statistical significance alone. (Changes added to the main document, page 13, lines 311-316)

3.9. Some adverse events take longer than others to occur and can be difficult to correlate (e.g., diabetes, weight gain) with the exposure. Particularly when evaluating a patient cohort with all hospitalized patients, there may be many reasons other than high-dose antipsychotics responsible for weight gain. For example, critically ill patients may be exposed to high-dose antipsychotics and gain weight related to critical illness rather than high-dose antipsychotics. Further, how will outcome variables such as weight gain or increased blood pressure be defined? Will it be defined as a single higher weight or blood pressure measurement or will to be a defined as a composite variable?

Answer: We recognise that outcomes such as weight gain or elevated blood pressure may result from factors other than high-dose antipsychotics, particularly in hospitalized patients. To address this, weight gain will be defined as an increase of ≥5% from baseline, and high blood pressure will require repeated measurements above standard clinical thresholds. Where appropriate, composite measures will be used to capture clinically meaningful changes and reduce misclassification, ensuring that outcome definitions are clear, consistent, and clinically relevant. We will adjust for potential confounding factors, including critical illness, comorbidities, and other relevant variables, to strengthen the validity of the independent association between the exposure and study outcomes. However, we acknowledge that, given the observational nature of the study design, the influence of unmeasured confounders cannot be fully excluded. (Changes added to the main document, page 12, lines 289-311)

VERSION 2 - REVIEW

**Reviewer**	**1**
**Name**	**Elhusein, Bushra**
**Affiliation**	**Hamad Medical Corporation, Psychiatry**
**Date**	**16-Sep-2025**
**COI**	

This is a well-written and thoughtfully designed study protocol addressing an important and timely issue: high-dose antipsychotic prescribing in acute hospital settings. The rationale is clearly articulated, the methods are comprehensive, and the planned analyses are excellent.

The protocol provides clear operational definitions for high-dose prescribing (both chlorpromazine equivalents and %BNF dose), which improves transparency and reproducibility.

However, I have a few points that would need clarification/expansion, by the authors.

1/ While dissemination plans include lay summaries, it would strengthen the protocol to acknowledge the lack of PPI in the design phase and reflect on the potential limitations of this. Even a short statement on how PPI could be integrated in future iterations or dissemination would be valuable.

2/ Several outcomes rely on proxy indicators (e.g., medication prescribing for extrapyramidal symptoms, cognitive decline). While this is understandable given the nature of routine data, it may be helpful to provide more detail on how the team will address potential misclassification bias, for example, sensitivity analyses or validation against subsets with more detailed clinical information.

3/ The integration of machine learning is exciting but perhaps under-described. It may help the reader to have more clarity on:

Which specific algorithms will be prioritized (beyond random forest)?

How will model performance be evaluated (AUROC, calibration, etc.)?

How will interpretability be balanced with predictive performance (e.g., use of SHAP values or feature importance metrics)?

4/ As HEPMA covers around 65% of the Scottish population, could the authors elaborate on whether prescribing practices in the uncovered boards differ systematically, and how that may limit generalizability?

5/ The abstract is concise and straightforward, but it may help to explicitly state that the study will not attempt to establish causality (given the retrospective design).

6/ Consider a sentence acknowledging potential secular trends (e.g., changes in guidelines or antipsychotic availability between 2019–2023) that may influence prescribing patterns.

7/ The manuscript uses polytherapy; however, the more common term in psychiatric pharmacology is antipsychotic polypharmacy. I believe using the latter (or clarifying equivalence) would improve clarity and consistency.

I believe addressing these points will add to the clarity of the protocol

Thank you!

**Reviewer**	**3**
**Name**	**Jaworska, Natalia**
**Affiliation**	**University of Calgary, Critical Care Medicine**
**Date**	**20-Sep-2025**
**COI**	

Thank you for the opportunity to review this manuscript again. The answers provided by the authors are generally responsive to reviewer comments. One minor comment remains around the reporting of outcomes from clinical notes. The authors have replied that outcomes from clinical notes will not be included in the study. However, it distinctly states in Table 2 under Psychiatric disease control that this outcome will be operationalized from "clinical notes and ICD-10 codes". This should be revised if clinical notes are not intended to be used by the authors for outcome measurements.

VERSION 2 - AUTHOR RESPONSE

Reviewer: 1
Dr. Bushra Elhusein, Hamad Medical Corporation
Comments to the Author:
This is a well-written and thoughtfully designed study protocol addressing an important and timely issue: high-dose antipsychotic prescribing in acute hospital settings. The rationale is clearly articulated, the methods are comprehensive, and the planned analyses are excellent.

The protocol provides clear operational definitions for high-dose prescribing (both chlorpromazine equivalents and %BNF dose), which improves transparency and reproducibility.

However, I have a few points that would need clarification/expansion, by the authors.

1/ While dissemination plans include lay summaries, it would strengthen the protocol to acknowledge the lack of PPI in the design phase and reflect on the potential limitations of this. Even a short statement on how PPI could be integrated in future iterations or dissemination would be valuable.

Answer: Following sentence has been added to the manuscript in PPI section 2.3 – "In future iterations, PPI groups will be involved in developing dissemination materials, including lay summaries, to improve accessibility and relevance of findings." (Changes added to the main document, page 18, lines 357-359)

2/ Several outcomes rely on proxy indicators (e.g., medication prescribing for extrapyramidal symptoms, cognitive decline). While this is understandable given the nature of routine data, it may be helpful to provide more detail on how the team will address potential misclassification bias, for example, sensitivity analyses or validation against subsets with more detailed clinical information.

Answer: Following sentence has been added to the manuscript – "Where outcomes rely on proxy indicators (e.g., medication prescribing for extrapyramidal symptoms or cognitive decline), we will conduct sensitivity analyses in subsets with more detailed clinical information, compare findings across multiple proxy definitions, and discuss potential misclassification bias in the interpretation of results." (Changes added to the main document, page 17, lines 339-343)

3/ The integration of machine learning is exciting but perhaps under-described. It may help the reader to have more clarity on:
Which specific algorithms will be prioritized (beyond random forest)?
How will model performance be evaluated (AUROC, calibration, etc.)?
How will interpretability be balanced with predictive performance (e.g., use of SHAP values or feature importance metrics)?

Answer: Following sentence has been added to the manuscript – "Random forest models will serve as the primary method, with gradient boosting (XGBoost, LightGBM) and regularised regression (LASSO, elastic net) included for comparison. Model performance will be assessed using AUROC, calibration metrics (Brier score, calibration plots), and interpretability through SHAP (SHapley Additive exPlanations) values and feature importance. To address potential confounding when comparing dose groups, propensity score matching and weighting methods will also be applied." (Changes added to the main document, page 18, lines 345-351)

4/ As HEPMA covers around 65% of the Scottish population, could the authors elaborate on whether prescribing practices in the uncovered boards differ systematically, and how that may limit generalizability?

Answer: At the time of the protocol submission, HEPMA coverage extended to approximately 65% of the Scottish population. Since then, coverage has expanded substantially, now reaching around 90% of the population. This greatly reduces concerns about systematic differences between covered and uncovered boards and enhances the generalisability of our findings. (Changes made to the supplementary document Table1)

5/ The abstract is concise and straightforward, but it may help to explicitly state that the study will not attempt to establish causality (given the retrospective design).

Answer: Thank you for this helpful suggestion. Following sentence added to the manuscript– "This study will describe prescribing patterns and associated outcomes but will not attempt to establish causality." (Changes added to the main document, page 2, lines 46-47)

6/ Consider a sentence acknowledging potential secular trends (e.g., changes in guidelines or antipsychotic availability between 2019–2023) that may influence prescribing patterns.

Answer: Following sentence has been added to the manuscript- "We also acknowledge that potential secular trends, such as changes in clinical guidelines or antipsychotic availability between 2019-2023, which may have influenced prescribing patterns." (Changes added to the main document, page 6, lines 136-138)

7/ The manuscript uses polytherapy; however, the more common term in psychiatric

pharmacology is antipsychotic polypharmacy. I believe using the latter (or clarifying equivalence) would improve clarity and consistency.

Answer: As explained previously, in our manuscript the term polytherapy is intentionally used to describe the concurrent prescribing of more than one antipsychotic medication. We recognise that antipsychotic polypharmacy is the more widely used term in psychiatric pharmacology; however, we believe polytherapy is more precise in the context of our study, as it avoids conflation with broader definitions of polypharmacy (i.e., multiple drugs across therapeutic classes). In addition, where the discussion extends beyond antipsychotics (e.g., in the section on covariates and confounders, page 16, line 301), we have retained the term polypharmacy to reflect the broader pharmacological context. (No changes made to the main document)

I believe addressing these points will add to the clarity of the protocol

Thank you!

Reviewer: 3
Dr. Natalia Jaworska, University of Calgary
Comments to the Author:
Thank you for the opportunity to review this manuscript again. The answers provided by the authors are generally responsive to reviewer comments. One minor comment remains around the reporting of outcomes from clinical notes. The authors have replied that outcomes from clinical notes will not be included in the study. However, it distinctly states in Table 2 under Psychiatric disease control that this outcome will be operationalized from "clinical notes and ICD-10 codes". This should be revised if clinical notes are not intended to be used by the authors for outcome measurements.

Answer: Thank you for pointing this out. This has been updated to “ICD10 codes” in the Table2. (Changes added to the main document, page 14, Table 2)

VERSION 3 - REVIEW

**Reviewer**	**1**
**Name**	**Elhusein, Bushra**
**Affiliation**	**Hamad Medical Corporation, Psychiatry**
**Date**	**16-Nov-2025**
**COI**	

Thank you for sending me the revised version. I can see that the authors carefully considered the earlier feedback and made the appropriate adjustments. I just wanted to let you know that I have no additional comments or concerns.
